# Moral disengagement in psychiatric inpatient care for individuals who engage in self-injury: a conceptual framework for understanding staff responses and patient experiences

**DOI:** 10.3389/fpsyg.2026.1900173

**Published:** 2026-09-10

**Authors:** Jonas Bjärehed, Marlene Bjärehed

**Affiliations:** 1 Department of Psychology, Lund University, Lund, Sweden; 2 Faculty of Education, Kristianstad University, Kristianstad, Sweden

**Keywords:** invalidating behaviors, moral disengagement, moral distress, organizational culture, psychiatric inpatient care, reflective support, self-injury behavior

## Abstract

Psychiatric inpatient care for individuals who engage in self-injury involves tensions between preventing harm, respecting autonomy, sustaining relational care, and managing organizational demands. Staff may experience emotional burden and moral distress, while patients may experience care as invalidating, coercive, or insufficiently responsive to their needs. This article develops a theory-informed conceptual framework applying Albert Bandura's theory of moral disengagement to psychiatric inpatient care for self-injury. Drawing on an interpretive synthesis of Bandura's theory and clinical literature identified through a focused, non-systematic search, it examines how moral disengagement-related processes may shape the interpretation, justification, and normalization of problematic care practices. The framework maps Bandura's eight moral disengagement mechanisms across four loci of moral self-regulation: conduct, agency, consequences, and recipients. It illustrates how practices may be interpreted as protective, reframed through euphemistic language, or made acceptable through comparison with more severe alternatives. Responsibility may be displaced onto institutional authority or diffused across teams, while the interpersonal and psychological consequences of care may be minimized or treated as secondary to immediate safety demands. At the recipient locus, patients may be dehumanized through reduction to diagnoses, risk profiles, or behavioral categories, or blamed for their self-injury and for the emotional and organizational burdens associated with care. The framework situates these mechanisms within a recursive multilevel process in which staff appraisals, care practices, patient responses, and team and organizational conditions may mutually shape one another over time. The analysis is conceptual and hypothesis-generating, not an empirical demonstration that moral disengagement has occurred in specific situations. Rather than locating problematic care primarily in individual failure, the framework directs attention to the relational, organizational, and societal conditions under which ethical responsiveness may be weakened or restored. Future research should operationalize these processes and examine them across staff and patient perspectives and individual and collective levels.

Findings across systematic reviews indicate that inpatient psychiatric care for people who engage in self-injury can be experienced by staff as emotionally demanding and difficult to manage, with fear of further injury and challenges in sustaining a person-centered therapeutic relationship (Karman et al., 2015; O'Connor and Glover, 2017). Patients' accounts have similarly described how negative or dismissive staff responses, poor communication, coercive practices, and limited recognition of individual needs can make care feel invalidating or unsafe (Butterworth et al., 2022; Lindgren et al., 2018; Scholes et al., 2022; Taylor et al., 2009).

Neither the emotional burden described by staff nor the negative care experiences reported by patients should be understood primarily as evidence of deficient professional practice or malicious intent. Rather, these experiences can be understood in relation to the complex clinical and organizational conditions in which inpatient care for self-injury is provided.

Against this background, we propose that moral processes, as conceptualized in Bandura's (2016) theory of moral disengagement, may offer a useful analytical framework for understanding how staff responses and patient experiences are interpreted, justified, and normalized within this complex care context.

## Self-injury and its ethical tensions in inpatient care

Self-injurious behavior, typically defined as the deliberate destruction of one's own body tissue without suicidal intent (Nock, 2009), is clinically understood as a maladaptive strategy for regulating overwhelming emotional states or interpersonal distress. Individuals with recurrent or severe self-injury are not infrequently treated within psychiatric inpatient care, where treatment can involve coercive measures, restrictions, and intensive monitoring in response to acute safety concerns and elevated suicide risk (Bowers, 2014). Under such conditions, emotional demands, risk-focused decision-making, limited resources, and insufficient staff support may affect both how staff experience their work and how patients experience the care they receive (Karman et al., 2015; O'Connor and Glover, 2017; Scholes et al., 2022; Butterworth et al., 2022).

Staff must navigate tensions between respecting patients' autonomy and preventing further injury, providing relational care and managing risk, and recognizing distress while responding to repeated or escalating crises (James et al., 2012; O'Connor and Glover, 2017). These tensions can make inpatient care of self-injuring individuals a particularly complex and demanding clinical intervention, especially when staff experience fear, helplessness, emotional burden, or limited therapeutic effectiveness (Nordkamp et al., 2024; O'Connor and Glover, 2017).

Adding to this complexity, directive, behavioral, or restrictive interventions can often be necessary in these situations. Heightened observation during periods of acute risk, clear and individualized behavioral boundaries, and temporary restrictions intended to prevent immediate injury may be clinically and ethically justifiable (O'Connor and Glover, 2017; Butterworth et al., 2022). Evidence-based treatments, such as Dialectical Behavior Therapy (DBT), may also incorporate contingency-based strategies, including efforts to avoid inadvertently reinforcing self-injurious behavior. In DBT, however, such strategies are combined with validation and acceptance of the person's emotions and experience. The aim is collaborative skill-building and increased agency rather than obedience, punishment, or withdrawal of care (Chapman, 2006). Contingency-based strategies, behavioral boundaries, and temporary restrictions should therefore not be regarded as problematic solely by virtue of their form. Their clinical and ethical significance depends on whether they are transparently linked to an explicit treatment rationale, implemented proportionately, and accompanied by continued recognition of the patient's distress, dignity, and personhood. Such practices may become ethically problematic when these conditions are absent, particularly when clinical rationales obscure avoidable distress, reduce recognition of the patient's subjectivity, or foreclose reflection on less restrictive alternatives.

## Understanding staff and patient experiences

Existing literature has examined staff experiences and patient experiences of care from several complementary perspectives. Research on healthcare professionals' attitudes has examined how self-injury may be interpreted in clinical practice, with negative attitudes reported across a range of healthcare settings (Karman et al., 2015). Research on organizational and ward-level conditions has examined how the context of psychiatric inpatient care may shape conflict, containment, and the management of self-injury (Bowers, 2014; O'Connor and Glover, 2017; Butterworth et al., 2022). Taken together, these perspectives raise two related but distinct questions: how conditions of care shape staff members' occupational and emotional experiences, and how those conditions may influence the moral reasoning through which care practices are interpreted, justified, or normalized.

Beyond research specifically focused on staff attitudes and organizational conditions, a growing body of literature has also examined other forms of staff burden. A systematic review of nurses' experiences of caring for inpatients with borderline personality disorder and/or self-injury described frustration, powerlessness, emotional exhaustion, and a risk of compassion fatigue arising from the unexpected demands of this work (Nordkamp et al., 2024). In a study of psychiatric nurses, burnout was associated with greater antipathy toward self-injury, whereas compassion satisfaction was associated with lower antipathy (Streeto and Phillips, 2024). Research on acute psychiatric nursing further shows that moral distress may arise when coercion, limited resources, staffing pressures, and other organizational constraints prevent nurses from providing care in accordance with their professional ideals (Jansen et al., 2020). These perspectives address related but distinct dimensions of the clinical context: burnout concerns prolonged occupational strain; compassion fatigue concerns the emotional effects of sustained exposure to patients' suffering and care demands; moral distress concerns ethical conflict under conditions of constrained agency. Together with research on negative evaluative beliefs and judgments associated with self-injury, these perspectives provide an emerging framework for understanding how emotional burden, relational difficulties, and ethical tensions may arise in the care of people who engage in self-injury.

Together, these perspectives highlight the emotional and ethical complexity of care for people who self-injure, but they do not fully explain how problematic responses may come to appear reasonable or necessary. Bandura's theory of moral disengagement offers a complementary framework for examining how such responses may be cognitively reframed, socially reinforced, and gradually normalized.

## The possible importance of moral processes

Moral disengagement refers to psychological and social mechanisms through which moral self-sanctions become weakened or selectively disengaged, allowing conduct that would otherwise conflict with internalized ethical standards to appear justified, necessary, or professionally appropriate (Bandura, 2016). The theory emphasizes how ordinary people may gradually participate in problematic practices when cognitive, emotional, interpersonal, and institutional conditions make those practices appear acceptable or necessary.

Although moral disengagement theory has been extensively applied within areas such as aggression (Gini et al., 2014), bullying (Killer et al., 2019), military violence (McAlister et al., 2006), and organizational misconduct (Newman et al., 2020), the framework may also be relevant to healthcare contexts (Moore, 2015). Applications of moral disengagement theory in healthcare to date remain relatively limited and have been concentrated primarily in general nursing research. However, emerging studies have begun to examine how nurses may cognitively justify departures from professional ethical standards in demanding clinical environments. Fida et al. (2016) developed and validated a measure of nurse moral disengagement and identified associations with counterproductive work behavior. More recent qualitative and quantitative studies among emergency and intensive-care nurses have also examined similar processes, such as justification, cognitive reconstruction, commitment avoidance, and the normalization of ethically problematic practices (Ke and Li, 2025; Khanipour-Kencha et al., 2025; Talebian et al., 2025). However, the application of the broader moral disengagement theory to psychiatric inpatient care—and particularly to the care of people who engage in self-injury—appears to remain unexplored.

### Aim and conceptual approach

The present article develops a theory-informed conceptual analysis that maps Bandura's mechanisms of moral disengagement onto phenomena described in the literature on psychiatric inpatient care for individuals engaging in self-injurious behavior. To identify empirical contexts and relevant primary studies, we conducted a focused, non-systematic literature search using http://elicit.com (accessed August 2026). The searches combined terms related to self-injury and self-harm, psychiatric inpatient care, staff and patient experiences, and organizational conditions. Systematic reviews were prioritized as entry points and supplemented by relevant qualitative and quantitative primary studies. The literature search was intended to provide empirical anchoring for the analysis rather than exhaustive coverage of the field. Relevant publications were selected according to their conceptual and clinical relevance to inpatient care for self-injury. The conceptual framework was developed through an interpretive synthesis of Bandura's (1999, 2002, 2016) theory of moral disengagement, the identified literature, and the authors' prior knowledge and clinical experience of the field. This expertise informed the conceptual focus and interpretation of candidate mechanisms, but was not treated as independent empirical evidence. The resulting framework should therefore be understood as a hypothesis-generating conceptual account rather than a systematically derived or empirically validated model.

Drawing on this literature, the article outlines the core principles of moral disengagement theory, examines how its mechanisms may manifest in inpatient psychiatric settings, and situates these processes within interactions among staff experiences, patient responses, team dynamics, and organizational structures. It then proposes a multilevel, recursive conceptual model in which staff appraisals, care practices, patient responses, and team and organizational conditions may mutually shape one another over time. The model is presented as a hypothesis-generating account for empirical investigation. Finally, it discusses limitations of the analysis and implications for future research and clinical practice.

More broadly, the article proposes moral disengagement as an integrative framework for understanding how emotionally demanding, organizationally strained, and morally complex care environments may contribute to problematic responses becoming morally justified, socially reinforced, and gradually normalized. Rather than locating such dynamics primarily within individual professionals, the framework emphasizes the interaction between psychological, relational, organizational, and societal processes operating across systems of care. Although the analysis focuses specifically on psychiatric inpatient care for self-injurious behavior, the framework may also be relevant to other psychiatric and healthcare settings characterized by prolonged exposure to patient suffering, recurrent crises, and tensions between care, control, and professional responsibility.

## Moral disengagement theory

Moral disengagement theory conceptualizes human behavior as shaped by reciprocal interactions between personal, behavioral, and environmental factors (Bandura, 2016; Moore, 2015). Building on Bandura's broader social-cognitive theory, it applies this interactionist and self-regulatory account of human agency to moral conduct and its regulation (Bandura, 1999, 2002). Moral conduct is regulated not only through external rules and professional standards, but also through processes of self-monitoring, self-evaluation, and anticipated self-sanctions such as guilt, shame, and self-condemnation. Hence, moral self-regulation is neither fixed nor continuously active. Under particular social and psychological conditions, moral self-sanctions that would ordinarily inhibit harmful conduct may be selectively disengaged (Bandura, 2002). Through moral disengagement, actions may then be perceived as acceptable, justified, necessary, or inconsequential (Bandura, 1999, 2016).

Bandura organized the mechanisms of moral disengagement around four loci within the self-regulatory process (Bandura, 2002). The first locus concerns conduct and includes (1) moral justification; (2) euphemistic labeling; and (3) advantageous comparison. The second concerns agency and includes (4) displacement of responsibility and (5) diffusion of responsibility. The third concerns consequences and includes (6) distortion or minimization of consequences. The fourth locus concerns the recipient of conduct and includes (7) dehumanization and (8) attribution of blame.

This organization is useful for the present analysis because it distinguishes between different ways in which moral responsiveness may be weakened. For example, a response may be framed as serving a valued purpose, attributed to institutional rules, understood as having insignificant consequences, or directed toward a patient who is perceived as responsible for or deserving of the response. These mechanisms are not assumed to operate independently; rather, they may interact and may be influenced by teams and organizations, within which interpretations can be reinforced through professional language, routines, leadership practices, and shared understandings of particular patients or groups. In this article, we propose that the complex clinical, relational, and institutional conditions characterizing psychiatric inpatient care for self-injury may increase the appeal of narratives that reduce emotional ambiguity and moral discomfort among staff. Simplified interpretations emphasizing manipulation, behavioral control, institutional necessity, or personal responsibility for the behavior may offer temporary psychological coherence and reduce ambiguity. However, the argument is not that individuals who engage in self-injury inherently evoke negative responses, nor that moral disengagement is inevitable within inpatient care. Rather, self-injury can create unusually strong pressures toward disengagement processes within systems lacking sufficient reflective, relational, and organizational support.

## Mechanisms of moral disengagement in psychiatric inpatient care

The following sections examine how these mechanisms might manifest in psychiatric inpatient care for self-injury and compare their classical formulations with analogous processes described in the clinical literature. Table 1 summarizes these illustrative parallels.

**Table 1:** Illustrative clinical expressions of moral disengagement mechanisms in psychiatric inpatient care.

Locus	Mechanism		Illustrative expressions in inpatient care
Conduct	Moral justification	Conduct is framed as necessary for worthy ends (e.g. protecting society, maintaining order).	Restrictive or coercive interventions are framed as necessary for patient protection, ward safety, or therapeutic structure. Emotional distancing is justified as necessary to avoid reinforcing self-injury or compromising professional functioning.
	Euphemistic labeling	Conduct is obscured through sanitized, technical, or bureaucratic language.	Distressing or coercive interventions are described primarily through procedural, diagnostic, or risk-management terminology that obscures their emotional impact on the patient. Emotionally distant approaches couched in therapeutic jargon.
	Advantageous comparison	Conduct is justified through comparison with more extreme forms of behavior.	Restrictive or emotionally distancing responses are perceived as acceptable because they fall short of more extreme coercive practices. Harsh or controlling interactions may appear justified when contrasted with more severe forms of coercion or restraint.
Agency	Displacement of responsibility	Agency is reduced when responsibility for one's actions is shifted to those who direct or authorize them.	Restrictive or invalidating practices are understood as mandated by institutional policy, safety procedures, or clinical hierarchies rather than as personally chosen actions.
	Diffusion of responsibility	Agency is reduced when responsibility for one's actions becomes dispersed across a group, reducing individual accountability.	Responsibility for interventions, or empathic responding, becomes dispersed across staff members, and shifts. Accountability becomes collective and diffuse, personal accountability is reduced.
Consequences	Distortion or minimization of consequences	Harmful consequences of conduct are underestimated because they are not fully perceived or psychologically appreciated.	The relational and emotional consequences of coercion, invalidation, or interpersonal distancing are underestimated or overlooked as each encounter appears minor or isolated. Short-term success of interventions may overshadow less visible but cumulative psychological and relational harms.
Recipient	Dehumanization	Individuals are reduced to categories, objects, or problems rather than recognized as full persons.	Patients with recurrent self-injury are understood primarily through diagnoses, risk profiles, behavioral concerns, or repeated crises rather than as persons with complex emotional experiences and histories.
	Attribution of blame	Individuals are perceived as responsible for the harm they experience because they allegedly provoked, caused, or deserved it.	Patients who engage in self-injury are viewed as responsible not only for their injuries but also for staff frustration, disruption within the ward, or strain on institutional resources.

These examples are conceptual applications of Bandura's mechanisms to psychiatric inpatient care. They are intended to clarify the framework and do not imply that moral disengagement has been empirically established in any specific situation.

The mechanisms are presented as hypotheses about how care may be interpreted, justified, and sustained, not as claims that particular interventions or staff responses demonstrate moral disengagement in themselves. Restrictive or directive interventions, clinical formulations, professional distance, and shared care processes may all be clinically appropriate and ethically justified. The relevant distinction concerns how such practices are justified and enacted: whether they serve a clearly specified and proportionate purpose, remain responsive to the patient's subjectivity and dignity, and remain open to communication, reflection, and review. Moral disengagement becomes relevant when clinical rationales make avoidable distress, loss of autonomy, or diminished recognition of the patient's personhood appear necessary, insignificant, or beyond moral scrutiny.

To make these hypotheses more concrete, each subsection includes two brief stylized formulations of how the relevant reasoning might be expressed in inpatient care. These formulations are illustrative constructions rather than verbatim quotations or reported staff statements.

### Moral justification

Moral justification refers to the cognitive reframing of potentially harmful conduct as serving a morally valued purpose. In Bandura's account, linking an action to a valued end can make it appear compatible with one's moral standards and reduce self-sanction for its harmful aspects. In psychiatric inpatient care for self-injury, this mechanism may be relevant when restrictive or coercive responses are understood primarily as necessary to protect life, prevent injury, maintain ward safety, or fulfill a duty of care. Such interventions are not inherently expressions of moral disengagement. The proposed mechanism becomes relevant when the protective purpose is treated as sufficient to settle the moral status of the intervention, thereby reducing attention to its proportionality, patient-experienced effects, and less restrictive alternatives.

Such reasoning might be expressed in stylized form as “We have to do this to keep the patient safe” or “If it prevents another injury, it is justified.” Close or constant observation provides a possible example. People receiving psychiatric care for self-injury have described constant supervision as coercive and emphasized dialogue, participation, predictability, and support for self-responsibility (Gerle et al., 2019). Similarly, staff may understand a practice as legitimate negotiation while patients experience it as coercive (Bendall et al., 2022). These findings do not establish staff members' motives, but they identify situations in which an intervention can be framed as protective while being experienced as harmful. The same tension may arise in other safety-oriented practices, such as restricting access to means, limiting activities, or imposing conditions after an episode of self-injury.

Moral justification is therefore hypothesized when preventing self-injury is treated as sufficient to make an intervention morally unproblematic, leaving limited room to consider its relational and psychological effects or whether less restrictive alternatives are available. This distinction is important because prevention-focused care may address immediate physical risk without necessarily addressing the distress that self-injury serves to regulate (Thomas and Haslam, 2017).

### Euphemistic labeling

Euphemistic labeling refers to the use of language that reconstructs potentially harmful conduct in less troubling or more acceptable terms. In Bandura's account, replacing morally charged descriptions with technical or neutral language can reduce attention to the interpersonal consequences of an intervention. In psychiatric inpatient care for self-injury, terms such as therapeutic neutrality, containment, and observation may have legitimate clinical meanings. They may function euphemistically, however, when a procedural or benign description substitutes for a concrete account of what the intervention requires of, or does to, the patient.

For example, an intervention described as observation may be experienced as intrusive supervision, negotiation as coercive, and limit-setting as invalidating or relationally distant. In stylized form, this may be expressed as “It is therapeutic observation, not coercive monitoring” or “We are setting limits, not punishing the patient.” People receiving inpatient psychiatric care for self-injury have described constant supervision as profoundly coercive, while staff may understand comparable practices as legitimate forms of negotiation or therapeutic management (Bendall et al., 2022). These discrepancies do not establish that staff members used language deceptively or intended to obscure harm. They identify situations in which clinical terminology may narrow moral attention to an intervention's presumed therapeutic function while making its patient-experienced effects, proportionality, and less restrictive alternatives less visible.

### Advantageous comparison

Advantageous comparison refers to a process through which potentially problematic conduct is made to appear less objectionable by contrasting it with a more extreme or restrictive alternative. In psychiatric inpatient care for self-injury, an intervention may therefore be framed as acceptable because it is less restrictive than restraint, seclusion, forced medication, or another feared outcome. Such comparisons can be clinically relevant when they form part of a genuine least-restrictive assessment. The proposed mechanism becomes relevant when being less severe than another option is treated as sufficient evidence of acceptability, rather than prompting continued consideration of whether the intervention is necessary, proportionate, and responsive to the patient's experience.

In stylized form, the reasoning might be expressed as “At least this is less restrictive than placing the patient in restraints” or “If we do not use close observation, we may have to resort to seclusion.” Close observation may thus be presented as preferable to more visibly coercive measures, although patients have described constant supervision as psychologically harmful and profoundly coercive (Gerle et al., 2019). Similarly, research on restrictive practices has documented psychological effects, poor communication, and perceived loss of human rights (Butterworth et al., 2022). These findings do not imply that choosing a less restrictive intervention is itself morally problematic. Rather, advantageous comparison is hypothesized when comparison with a worse alternative narrows moral attention to what could have happened and makes the harms, proportionality, or avoidability of the intervention actually used less visible.

### Displacement of responsibility

Displacement of responsibility refers to a process through which individuals perceive their actions as determined by an authority, institutional rule, or external demand rather than by their own moral agency. Responsibility is thereby relocated upwards within a hierarchy, allowing conduct that conflicts with personal or professional values to be experienced as required or unavoidable. In psychiatric inpatient care for self-injury, this mechanism may be relevant when staff understand an intervention as required by a senior clinician, institutional policy, legal framework, or risk-management procedure.

The presence of policy, delegation, and formal guidance does not in itself demonstrate displacement of responsibility. Such structures may clarify roles and support safe and consistent care. The proposed mechanism becomes relevant when reference to institutional authority ends rather than supports moral deliberation. In stylized form, statements such as “I have no choice; those are the rules” or “The doctor decided it” may locate responsibility primarily in the institution and weaken the perceived connection between one's own conduct and the patient's experience. Acute psychiatric nurses have described coercion, constrained resources, and the gap between professional ideals and clinical reality as morally distressing, illustrating the kinds of conditions in which this distinction may become important (Jansen et al., 2020). The relevant question is whether such explanations foreclose reflection on how an intervention is implemented, whether it remains necessary and proportionate, and whether less harmful alternatives remain available.

Moral distress and institutional constraint provide an important context for displacement, but they do not demonstrate that staff have relinquished moral responsibility. The mechanism is hypothesized specifically when institutional authority is invoked in a way that treats patient suffering as an unavoidable consequence of the system rather than as an ethically salient feature of care requiring continued attention.

### Diffusion of responsibility

Diffusion of responsibility refers to a process through which accountability becomes distributed across a group, reducing the extent to which any individual experiences themselves as responsible for an ethically problematic outcome. Unlike displacement of responsibility, diffusion disperses responsibility laterally across staff members, professional roles, shifts, and procedures.

Psychiatric inpatient care is inherently team-based, and shared care plans, multidisciplinary discussions, handovers, and documentation are necessary features of safe and continuous care. Their presence does not in itself demonstrate diffusion of responsibility. The proposed mechanism becomes relevant when collective processes allow a plan or practice to be accepted without any individual or group taking responsibility for revisiting its effects on the patient. In stylized form, expressions such as “It was a team decision,” or “That is how the ward handles it” may indicate that collective agreement has replaced active ethical reflection. In a study of inpatient care for deliberate self-injury, nurses and psychiatrists linked clear roles, communication, shared expectations, and mutual respect to collaboration, and described greater autonomy and responsibility when roles were well defined (Löfström et al., 2025). The illustration becomes plausible where those safeguards fail—particularly because incomplete handover can leave clinicians without information needed to prioritize care and assess urgency (Davies and Enemo-Okonkwo, 2021).

Diffusion of responsibility may therefore contribute to the normalization of restrictive, emotionally distancing, or invalidating practices when no one retains clear moral ownership of the patient-experienced consequences of care. The mechanism concerns neither teamwork nor shared decision-making as such, but situations in which responsibility becomes so dispersed that potentially harmful interactions, stigmatizing interpretations, or established ward practices are not questioned or reviewed.

### Distortion or minimization of consequences

Distortion or minimization of consequences refers to the process through which the harmful effects of conduct are ignored, discounted, or represented as insignificant. In Bandura's account, underestimating the emotional, interpersonal, or practical impact of one's actions reduces the moral pressure to reconsider them. In psychiatric inpatient care for self-injury, this mechanism may be relevant when dismissive comments, emotionally distant interactions, or restrictive interventions are treated as secondary to the immediate tasks of managing risk, monitoring safety, or maintaining ward order. The proposed mechanism does not require that staff deny harm; consequences may instead be construed as transient, unavoidable, or insufficiently important to alter the intervention.

Repeated self-injury and recurrent crises may make an individual interaction appear minor when viewed as one episode among many. In stylized form, statements such as “Nothing serious happened; the patient eventually calmed down” or “It was only a few minutes, and there was no visible injury” may illustrate how the consequences of care are minimized. For patients, however, brief dismissive encounters, limited communication, or restrictive interventions may carry substantial psychological and relational significance, particularly when trust and therapeutic continuity are already weak. Research on restrictive practices has described them as potentially anti-therapeutic, dehumanizing, and punitive, and negative experiences of care after self-injury have also been linked to subsequent self-injury in emergency-care research (Griffin et al., 2025; MacDonald et al., 2020).

Distortion or minimization of consequences is therefore hypothesized when the absence of overt distress, the recurrence of crises, or the apparent success of risk containment is treated as evidence that an intervention has caused little interpersonal or psychological harm. The mechanism concerns how harms that are less visible than immediate physical injury become morally peripheral to the evaluation of care.

### Dehumanization

Dehumanization refers to a process through which individuals are perceived as lacking qualities associated with full personhood, individuality, or emotional complexity. In psychiatric inpatient care for self-injury, it is unlikely to appear in overt or extreme forms. It may instead involve a gradual reduction of patients to their diagnosis, self-injury, risk status, or perceived impact on ward functioning.

Stylized examples of dehumanization may be expressed as “We need to manage the risk, not engage with the person” or “The risk profile matters more than the patient's individual story.” Patient-focused research provides relevant contexts for examining this possibility. Research on restrictive practices has identified psychological effects, communication problems, and perceived loss of human rights as central features of patients' experiences (Butterworth et al., 2022). Interviews with women who had experienced restrictive interventions similarly identified dehumanization alongside powerlessness and problems in relationships and communication (Scholes et al., 2022). These findings document patient-experienced effects, but do not establish that staff consciously intended to dehumanize patients. The proposed mechanism becomes relevant when diagnostic, risk-based, or behavioral formulations become the dominant lens through which a patient is understood and treated.

In such situations, the patient's account, individuality, history, and preferences may receive less attention, while relational care is subordinated to behavioral control. Dehumanization is therefore hypothesized not as the use of clinical categories or structured safety measures in themselves, but as a process through which these formulations progressively displace recognition of the patient's subjectivity and suffering.

### Attribution of blame

Attribution of blame refers to a process through which responsibility for harmful treatment or its consequences is shifted onto the recipient. In psychiatric inpatient care for self-injury, the intentional character of the behavior may invite a conflation of agency with culpability. The proposed mechanism becomes relevant when self-injury is interpreted primarily as manipulative, attention-seeking, deliberately disruptive, or chosen by the patient.

In stylized form, this may be expressed as “They chose to do this, so they have to accept the consequences” or “They are responsible for the disruption they have caused on the ward.” Such interpretations may shift attention away from psychological distress and toward the patient's presumed intention, making reduced empathy, delayed engagement, or coercive responses appear more warranted. They may also position the patient as responsible for the emotional and organizational burden associated with recurrent admissions, staff time, or the use of ward resources. In this way, agency may be transformed into culpability, so that the patient is implicitly regarded as having brought restrictive, distancing, or invalidating care upon themselves.

Research indicating that the reasons for self-injury may be misconstrued, resulting in unhelpful responses and stigmatizing labels, provides a relevant empirical context for this hypothesis (Akinola and Rayner, 2022). Such findings do not establish that staff consciously blamed patients or that particular responses resulted from moral disengagement. They identify situations in which interpretations of responsibility may reduce recognition of distress and make the patient-experienced consequences of care less morally salient.

## Moral disengagement as a recursive multilevel process in psychiatric inpatient care

**Figure 1:**
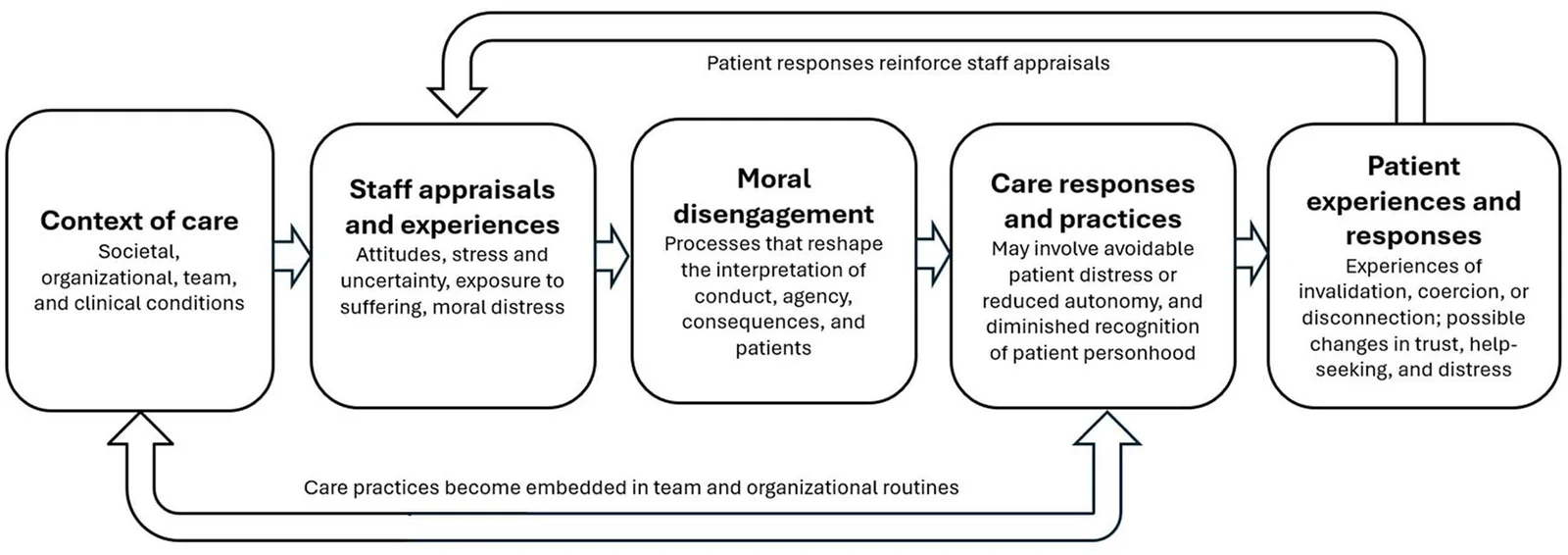
Proposed multilevel and recursive process through which moral disengagement-related mechanisms may emerge and become embedded in psychiatric inpatient care. Societal, organizational, team, and clinical conditions shape staff experiences and appraisals. These may influence how conduct, responsibility, consequences, and patients are interpreted, which may in turn shape care responses and patient experiences. Patient responses may reinforce staff appraisals, while repeated care practices may become embedded in team and organizational routines.

In this section, we integrate moral disengagement theory within a systems perspective (Figure 1). Consistent with Bandura's social-cognitive account of reciprocal interactions among personal, behavioral, and environmental factors, we propose that moral disengagement-related processes in psychiatric inpatient care are not determined by individual appraisals alone. Rather, we conceptualize these processes as emerging through interactions among conditions operating at different levels of the care system.

At the societal level, wider narratives concerning self-inflicted injury, intentionality, manipulation, responsibility, deservingness, and risk may inform how self-injury is understood before any individual clinical encounter occurs. At the organizational level, staffing, continuity of care, leadership, protocols, resource constraints, and institutional priorities may influence whether relational, reflective responses or control-oriented responses are practically supported and normalized. At the team level, staff appraisals may be reinforced, challenged, or modified through group processes, informal language, and local ward norms. At the clinical and individual level, the complexities of care, emotional burden, and moral distress may shape how a particular episode of self-injury and a particular patient are interpreted (Karman et al., 2015; Jansen et al., 2020; O'Connor and Glover, 2017). Within this multilevel context, moral disengagement mechanisms may operate as an interpretive link between staff experiences and care practices.

The process is proposed as recursive rather than linear. Staff experiences and appraisals may shape care responses and practices; patients may experience those responses as invalidating, coercive, disconnected, or unsafe, with possible effects on trust, help-seeking, distress, withdrawal, or dysregulation. These responses may then be interpreted by staff as resistance, disruption, non-engagement, or confirmation that more restrictive, controlling, or emotionally distant responses are required. At the same time, repeated care practices may become embedded in team and organizational routines, thereby altering the context in which subsequent episodes are understood and managed.

Figure 1 summarizes this proposed multilevel and recursive process. It should be read as a conceptual model for empirical investigation, not as a claim that the depicted pathways occur in every setting or follow a fixed causal sequence. Its purpose is to show how moral disengagement mechanisms may connect contextual conditions, staff appraisals, care practices, and patient experiences over time, and how the same system may also contain opportunities for interruption through reflective practice, relational continuity, collaborative communication, and organizational support.

## Future research directions

Future research should test the explanatory value of moral disengagement in psychiatric inpatient care and determine what it adds beyond related constructs, including burnout, compassion fatigue, moral distress, stigma, depersonalization, and moral injury. Studies should examine whether these constructs represent distinct, sequential, or interacting processes, and should identify empirical markers that distinguish moral disengagement from clinically appropriate practice and adaptive coping. This will require context-sensitive measures that capture not only individual beliefs but also their relational and institutional expression. Longitudinal and mixed-method studies should then examine how these processes develop over time and how they relate to observable care practices. Such studies should integrate staff and patient perspectives with observational or other behavioral indicators of care.

A further priority is to examine moral disengagement as a collective ward process (Schaefer et al., 2026). Multilevel studies combined with observational and qualitative inquiry could investigate collective moral disengagement, for example, how shared narratives and team norms are formed and reinforced through everyday interactions and leadership. Comparative research could also examine consultation-liaison psychiatry and medical psychology as potentially relevant contexts for investigating moral disengagement-related processes across psychiatric, emergency, and general hospital care. Intervention studies could then examine whether different practices, such as reflective supervision, trauma-informed and relationally continuous care, and psychologically supportive team cultures, reduce disengagement-related practices and strengthen moral engagement. This would shift the focus from identifying problematic attitudes in individual professionals to understanding how psychiatric organizations can prevent their normalization and sustain ethically engaged care (Bandura, 2016; Moore, 2015; Newman et al., 2020).

## Clinical and organizational implications

If moral disengagement is partly shaped by emotional overload, organizational strain, and shared interpretive practices, education about self-injury alone is unlikely to be sufficient to improve care. Educational interventions remain important, particularly when they include reflective and interactive components, but they should be combined with structures that support staff in managing distress, examining assumptions, and maintaining relational engagement (Karman et al., 2015).

At the professional and team levels, reflective supervision, psychologically safe discussion of difficult encounters, and opportunities to examine stigmatizing language or risk-focused assumptions may help preserve moral and relational responsiveness. At the organizational level, continuity of care, clear and reviewable procedures, adequate staffing, ethical leadership, and support following restrictive interventions may reduce the conditions under which responsibility is displaced, consequences are minimized, or distancing becomes normalized (O'Connor and Glover, 2017; Butterworth et al., 2022; Jansen et al., 2020).

The framework therefore shifts the focus of intervention from correcting individual attitudes alone to sustaining the conditions for ethical engagement across the ward.

## Limitations

Several limitations to the argument made in this article should be acknowledged. First, we present a theory-informed conceptual analysis rather than an empirical study or systematic review. The application of Bandura's mechanisms to psychiatric inpatient care is therefore interpretive and cannot establish whether moral disengagement occurs, how frequently it occurs, or whether it causes the staff responses and patient experiences discussed. The framework should consequently be understood as a hypothesis-generating model rather than a validated explanatory account.

Second, moral disengagement may be difficult to distinguish from adaptive professional coping, clinically justified boundaries, and necessary risk-management practices. Applying the framework too broadly could therefore pathologize staff responses or understate the emotional, organizational, and legal constraints under which care is provided.

Finally, the analysis focuses on severe self-injurious behavior in psychiatric inpatient settings, and its relevance to other patient groups, care contexts, and cultural or organizational settings remains untested. Future empirical research should examine the framework against alternative explanations and include both staff and patient perspectives.

## Conclusions

In this article, we have applied Bandura's theory of moral disengagement as a conceptual framework for examining how problematic responses to self-injury may become interpreted, justified, and normalized within psychiatric inpatient care. The framework directs attention to the moral processes through which ethically problematic practices may become compatible with professional and institutional narratives of safety, treatment, and necessity.

We have situated these processes within a multilevel and recursive system in which staff emotional burden, team narratives, organizational routines, patient responses, and broader assumptions about responsibility and deservingness may shape one another over time. The framework does not equate restrictive, behavioral, or risk-oriented care with moral disengagement, nor does it identify individual professionals as morally deficient. Rather, it concerns how practices may be morally constructed and sustained when patient subjectivity is reduced, responsibility is displaced or diffused, or consequences are minimized.

The practical implication is that ethical engagement cannot be sustained through correcting staff attitudes alone. Psychiatric organizations must also support reflective practice, relational continuity, psychologically safe team cultures, clear and reviewable procedures, and leadership attention to the emotional and moral demands of inpatient care.

Bandura's theory therefore offers a way to ask not only why care becomes difficult, but how particular responses come to appear necessary, acceptable, or beyond question. As a theoretical account, its value will depend on future research that examines these processes across individual and collective levels, includes both staff and patient perspectives, and tests them against alternative explanations.
